# Autophagy in Immune-Related Renal Disease

**DOI:** 10.1155/2019/5071687

**Published:** 2019-11-07

**Authors:** Xin Ye, Xu-jie Zhou, Hong Zhang

**Affiliations:** Renal Division, Peking University First Hospital, Peking University Institute of Nephrology, Key Laboratory of Renal Disease, Ministry of Health of China, Key Laboratory of Chronic Kidney Disease Prevention and Treatment (Peking University), Ministry of Education, Beijing 100034, China

## Abstract

Autophagy is an important biology process, central to the maintenance of biology process in both physiological and pathological situations. It is regarded as a “double-edged sword”—exerting both protective and/or detrimental effects. These two-way effects are observed in immune cells as well as renal resident cells, including podocytes, mesangial cells, tubular epithelial cells, and endothelial cells of the glomerular capillaries. Mounting evidence suggests that autophagy is implicated in the pathological process of various immune-related renal diseases (IRRDs) as well as the kidney that underwent transplantation. Here, we provide an overview of the pathological role of autophagy in IRRDs, including lupus nephritis, IgA nephropathy, membrane nephropathy, ANCA-associated nephritis, and diabetic nephropathy. The understanding of the pathogenesis and regulatory mechanisms of autophagy in these renal diseases may lead to the identification of new diagnostic targets and refined therapeutic modulation.

## 1. Introduction: Current Perspectives on the Pathogenesis of Immune-Related Renal Diseases

**Figure 1:**
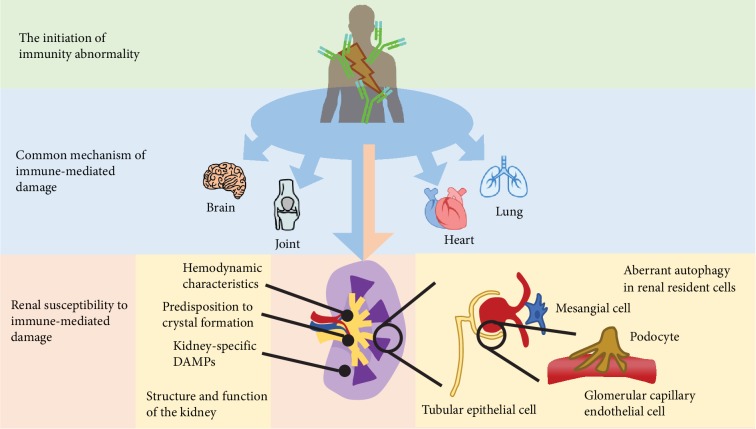
Immune-related renal disease. Aberrant immunity, such as autoimmune diseases, is a systemic disease. These immune disruptions, such as autoantibody production, immune complex formation, and disposition, can cause damage to any organ of our body, such as the heart, the lung, and the joints. However, the kidneys are susceptible to these immune-mediated damages, which results from its unique hemodynamic characteristics, kidney-specific DAMPs, and crystal formation in the tubule system. Besides, the renal resident cells, including podocytes, glomerular capillary epithelial cells, tubule epithelial cells, and mesangial, are also found to be susceptible to immune-mediated injuries.

Most immune-related renal diseases, or glomerulonephritis, frequently affect young people, often cannot be cured, and significantly lead to chronic kidney disease and end-stage renal failure, with associated morbidity and cost. In the past several years, there have been extensive researches focusing on its pathogenesis, which helps to gain increasing knowledge about cause and treatment. Traditionally, aberrant immunity is in the research spotlight for disease occurrence and progression and may also be relevant to other autoimmune diseases. Nevertheless, this organ is susceptible to various immunity-associated assaults, of which the underlying mechanisms are nowadays paid more attention to. Among these intriguing features, the process of autophagy in renal resident cells seems to serve as a protective role from certain injuries and toxic exposure, although research data are sometimes inconsistent (Table 1). The regulation and function of autophagy is likely cell type and context specific (Figure 1). Research into the role of autophagy in kidney physiology and pathogenesis still remains a largely understudied field.

## 2. Overview of Autophagy

Autophagy is a universal cell biology process in eukaryotic cells. It eliminates injured organelles and biological macromolecules. It is proven to be an important and highly conservative regulation mechanism to maintain intracellular homeostasis. Compared to the ubiquitin-proteasome system (UPS) that selectively degrades short-lived proteins, autophagy prefers aged and dysfunctional cytoplasmic proteins [1].

**Figure 2:**
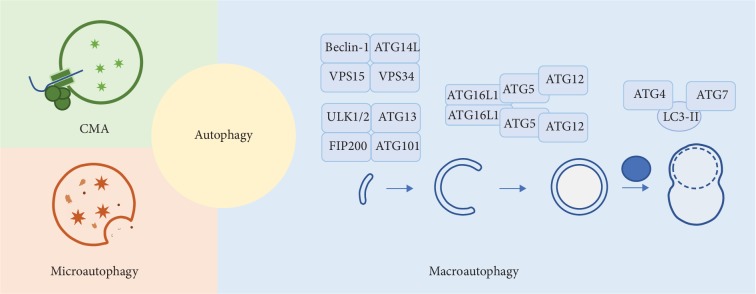
Classic autophagy pathway. There are three major types of classic autophagy process in mammalians: macroautophagy, microautophagy, and chaperone-mediated autophagy (CMA). Macroautophagy includes four major steps: induction, elongation, and maturation of autophagosome and fusion with lysosome. Several protein complexes are involved in this process.

In general, baseline autophagy in mammalian is a physiological process but can be triggered by starvation or by various conditions, including ischemic, toxic, immunological, and oxidative insults. The process of autophagy consists of two major steps: induction of autophagosome and fusion of autophagosome with lysosome (for detailed description, please refer to expert reviews, i.e., [2, 3]). A large number of autophagy-related (ATG) proteins are involved in the process of autophagy (Figure 2). ATG proteins can be divided into five groups, namely, ATG1 kinase complex (ATG1/Unc-51-like kinase (ULK) 1/2), ATG9, class III phosphoinositide 3-kinase complex (PI3KC3), ATG12 conjugation system, and ATG8 conjugation system [1].

## 3. Autophagy in Immune Cells

Autophagy in immune cells significantly alters immune activity. In innate immunity, autophagy achieves to augment immune cell activity and helps to fight against infection. Bacteria, viruses, and parasites can be degraded by autophagy, in which autophagy plays a protective role against these pathogens. Autophagy also restricts inflammation by interacting with certain signaling pathways and by engulfing inflammation triggers. In autophagic lysosomes, toll-like receptors (TLRs) might recognize damage-associated molecular pattern molecules (DAMPs) and pathogen-associated molecular pattern molecules (PAMPs) as autologous antigens and lead to an effective activation of immune reactions [4], which undermines immune tolerance and leads to the development of autoimmunity [5].

Several players in the innate immune system are regulated by autophagy to certain extent, such as macrophage and dendritic cell. Macrophage helps eliminate pathogens through intracellular digestion. Autophagy-deficient macrophage upregulates IL-18 and IL-1*β* production in the face of inflammatory stimulation through the TLR pathway [6]. Autophagy also contributes to caspase-independent macrophage cell death, which decreases inflammation [7]. In dendritic cells, autophagy is required for virus detection, antigen presentation, and inferon production [8, 9].

Several adaptive immune responses, such as lymphocyte development and antigen presentation, can be enhanced by autophagy activity [10]. Autophagy-mediated MHC class II presentation is a case in point. Extracellular antigens are captured into the autophagosomes of antigen-presenting cells. The autophagosome then degrades the antigens into immunogenic peptides and loads them onto MHC-II molecules to CD4+ T cells.

Accumulating evidence suggests that autophagy plays a pivotal role in T cell selection and survival. For example, in the selection of naïve T cell repertoires in the thymus, high autophagy activity in thymic epithelial cells achieves to deliver endogenous proteins to MHC-II molecules and contributes to TCR selection, consequently eliminating autoreactive CD4+ T cells [11]. Autophagy in activated T cells promotes survival and secretion of cytokines such as IL-2 and IFN-*γ*, thus influencing Th cell polarization.

As for B cells, autophagy plays a complex role. Unlike mature T cells, the survival of mature peripheral B cells seems not to necessarily require autophagy. On the one hand, autophagy is essential during B cell differentiation, i.e., *Atg5* deletion dramatically results in B-1 cell death [12]. On the other hand, autophagy can also induce autophagy-associated cell death [13]. Therefore, B cell receptor ligation-induced autophagy might be essential in eliminating self-reactive B cells, thereby reducing autoimmunity. In addition, recent data suggest that autophagy regulates ER homeostasis to control immunoglobulin (Ig) secretion in plasma cell, and yet deleting *Atg5* can lead to excessive Ig production [14].

## 4. Autophagy in Renal Resident Cells

### 4.1. Podocytes

Terminally differentiated podocytes are susceptible to various insults. From the perspective of pathology, the loss of podocytes is considered a key feature in progressive glomerular disease. Podocyte injury is the key factor in proteinuria, and loss of podocytes by cell death or detachment is a critical step for the progression of glomerular diseases and aging [15]. Autophagy appears to “monitor” the quality of podocytes under physiological and pathophysiological conditions. Podocytes from patients with minimal change disease (MCD) showed higher levels of Beclin 1-mediated autophagic activity than those from patients with focal segmental glomerulosclerosis (FSGS) [7, 16]. Furthermore, a high level of autophagy in podocytes of MCD patients often predicts a stable disease status, while MCD patients with decreasing levels of autophagy progressed to FSGS [17].

Loss of autophagy in podocytes results in a markedly increased susceptibility to various models of renal disease. Recent study showed that mice with *Atg5* or *Atg7* loss-of-function mutation developed histological and clinical characteristics of human FSGS. Silencing *Atg5* or *Atg7*, respectively, also showed significant podocyte alternations. One day postnephrectomy, mice with *Atg7*-deficient podocytes exhibited much higher proteinuria, as well as foot process effacement and podocyte loss in renal biopsy [18]. Podocyte-specific *Atg5* knockout mice developed mild proteinuria by 8–12 months of age and defunction to degrade damaged mitochondria via mitophagy [19]. Inhibiting autophagy by silencing other ATG genes also undermines podocyte functions. Mice with podocyte-specific deletion of *Vps34* developed early proteinuria, progressive glomerulosclerosis, and renal failure by 9 weeks [20]. Podocytes from these knockout mice displayed a phenotype of impaired autophagic flux with accumulation of enlarged vacuoles, suggesting that Vps34 participates in maintaining autophagic flux in podocytes. Similarly, podocyte-specific prorenin receptor- (*PRR*-) knockout mice developed nephritic syndrome within 2-3 weeks after birth and died by the 4th week. Electron microscopy revealed that the mice displayed progressive podocyte damage with foot process effacement and vacuolation and podocyte cell death [21, 22]. Dramatic accumulation of ubiquitinated protein and ubiquitin-binding scaffold protein p62/sequestosome 1 (SQSTM1) further suggested a block in autophagic clearance of the ubiquitinated protein aggregates [23]. Taken together, these studies highlight the particular importance of autophagy as a key homeostatic mechanism for podocytes under physiological and stress conditions.

Modulating mTOR to alter autophagy activity also influences podocyte structure and function. Podocyte-selective knockout of the *Mtor* gene mice developed proteinuria at 3 weeks of age with progressive podocyte damage and ultimately end-stage kidney failure by 5 weeks of age [24]. Similarly, in immortalized human podocytes, treatment with mTOR inhibitor rapamycin induced incomplete autophagy, showing a favorable cytoprotective effect. However, mTOR is also required to regenerate functional lysosomes and completion of autophagic process [25]. Thus, prolonged activation of mTOR may lead to lack of autophagy substrate, causing autophagy insufficiency. Given that disruption of the autophagic pathway may play a role in the pathogenesis of proteinuria, therapy with mTOR inhibitors can lead to both favorable and unfavorable consequences regarding to different time duration.

**Figure 3:**
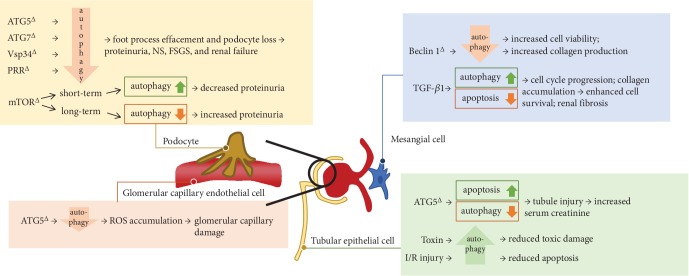
Autophagy in renal resident cells. The figure summarizes current studies of autophagy in renal resident cells, including podocytes, glomerular capillary epithelial cells, mesangial cells, and tubule epithelial cells. Upregulation or downregulation of autophagy activity through gene expression modulation or under certain stimulation can influence the survival of these cells and the overall function of the kidney. Taken together, autophagy plays a protective role in the physiology and pathophysiology of the kidney.

In short, autophagy is essential in podocyte survival and physiological functions. Blocking autophagy by inhibiting *ATG5*, *ATG7*, *mTOR*, *Vps34*, and *PRR* leads to podocyte cell injury, death, or dysregulated function and ultimately leads to decreased renal function (Figure 3).

### 4.2. Endothelial Cells of the Glomerular Capillaries

In a report of cultured cells from Fabry disease, cells from patients showed increased autophagy with a higher basal level of LC3. In the study, renal biopsies were obtained before and after 3 years of treatment. It showed that vacuole accumulation in endothelial cells and mesangial cells was drastically decreased after a 3-year therapy [26]. These data suggest that impairment in endothelial cell autophagic processes may contribute to Fabry disease in endothelial cell injury.

Recent study also demonstrated that autophagy protected glomerular endothelial cells in reacting to reactive oxidant species (ROS) [27]. Glomerulus endothelial and hematopoietic cell-specific *Atg5*-deficient mice presented with abnormal morphology in glomerulus and ROS accumulation, which can be attenuated by administration of ROS scavenger. These data suggest that autophagy in epithelial cells protects the glomerular capillary from oxidative stress and maintains its integrity.

### 4.3. Mesangial Cells

Glomerular mesangial cells are located in the centrilobular region called the mesangium, providing support for the glomerular structure as well as regulating glomerular filtration [28]. Mesangial cells also produce extracellular matrix that makes up the mesangium, in maintaining the homeostasis of kidney interstitial. However, they can also act as a deterioration factor in the development of a number of glomerular diseases, i.e., IgA nephropathy. When the kidney suffers from progressive kidney disease, mesangial cells proliferate and produce excessive extracellular matrix, leading to the development of glomerulosclerosis and kidney fibrosis.

It was observed that cadmium induced both autophagy and apoptosis in mesangial cells. But autophagy blockade resulted in increased cell viability without affecting apoptosis, suggesting that autophagy plays a role in cell death in mesangial cells exposed to cadmium [29]. It was reported that cadmium induced autophagic cell death through a calcium-extracellular signal-regulated kinase-dependent pathway and, in part, through increased reactive oxygen species (ROS) production and activation of glycogen synthase kinase-3b (GSK-3b) [30].

Autophagy also contributed to survival of mesangial cells. Under the condition of serum deprivation, mesangial cells undergo apoptosis. Transforming growth factor-*β*1 (TGF-*β*1) promoted autophagy and enhanced cell survival by inhibiting mesangial cells from undergoing apoptosis. LC3^−/−^ mesangial cells abrogated TGF-*β*1 rescue from serum deprivation-induced apoptosis, indicating a cytoprotective role of autophagy in mesangial cells [31]. Autophagy also plays a role in downregulating the production of matrix in mesangial cells by accelerating the process of degrading intracellular type I collagen (Col-I) produced by mesangial cells [31]. Beclin 1^+/-^ mice presented with significantly increased collagen deposition in the kidneys. Mesangial cells isolated from Beclin 1^+/-^ mice or transfected with Beclin 1 siRNA-expressed higher basal level of Col-I. Also, mesangial cells that were treated with an autophagy inhibitor showed an increased Col-I protein level. Accordingly, treatment with trifluoperazine, an inducer of autophagy, resulted in a decreased Col-I protein level induced by TGF-*β*1.

Consequently, it is suggested that autophagy may constitute an adaptive mechanism to glomerular injury by inhibiting apoptosis and promoting mesangial cell survival. The findings also implicate a novel role of autophagy as a cytoprotective mechanism to negatively regulate and prevent excess collagen accumulation in the glomeruli and hold promise for a new therapeutic target to mitigate pathogenesis of glomerulosclerosis and fibrosis.

### 4.4. Renal Tubular Epithelial Cell (TEC)

Unlike podocytes, tubules display a low level of basal autophagy under normal conditions. Mice with *Atg5* deletion in proximal tubules gradually developed deformed mitochondria and accumulation of cytosolic inclusions, leading to proximal tubular cell hypertrophy and eventual degeneration. Mice with *Atg5* deletion in distal tubules also displayed a significant accumulation of p62/SQSTM1 and oxidative stress markers, without significant alteration in kidney function up to 12 months of age [32]. *Atg5* deletion in the entire tubule system resulted in accumulation of p62/SQSTM1 throughout the tubular segments, and at 5 months, there was a significant increase in serum creatinine [32]. Therefore, while ATG5 deficiency solely in proximal or distal tubular cells did not cause significant renal dysfunction, ATG5 deficiency in all tubule segments caused impairment of kidney function, suggesting tubular autophagy is important in the preservation of kidney function.

In facing exposure to environmental toxins, activated autophagy is also observed in the tubular cells. Upregulation of autophagy before apoptosis was detected in the proximal tubules of mice injected with cisplatin. Inhibiting autophagy enhanced the activation of caspases and apoptosis in cisplatin-treated proximal tubular cells. It suggested that autophagy protected tubular cells from apoptosis [33]. Proximal tubule-specific autophagy-deficient mice developed more severe AKI and increased apoptosis after cisplatin treatment [34, 35]. Moreover, autophagy-deficient proximal tubules exhibited with increased DNA damage, p53 and c-Jun N-terminal kinase (JNK) activation, and accumulation of toxic protein aggregates and ROS after cisplatin treatment. Similar phenomenon is also observed when TECs are treated with aristolochic acid or cyclosporin A. Autophagy activity is activated when toxin is administrated, and suppressing autophagy induced a higher level of apoptosis [36–38].

In models of obstructive nephropathy induced by unilateral ureteral obstruction (UUO), it was observed that mTOR was inhibited and cell autophagy activity was enhanced in order to remove abnormal intracellular components. Similarly, autophagy is confirmed as a protective role in the face of renal I/R injury. It demonstrated that blocking autophagy enhanced hypoxia-induced apoptosis in cultured renal proximal tubular cells [35]. Studies using mice with conditional *Atg5* or *Atg7* gene deletion in the proximal tubule confirmed that autophagy protected the proximal tubule from I/R injury. These results provide further support for the cytoprotective role of autophagy in the tubules.

In other circumstances, autophagy in the tubules serves as a contributing factor to the disease development. Another UUO model in mice revealed that autophagy is associated with renal fibrosis. Inhibiting autophagy resulted in suppressed renal fibrosis through downregulating profibrotic factors [39]. Nephropathic cystinosis is one of the lysosomal storage diseases that characterized with an abnormal function of the kidney tubules and progressive development of renal insufficiency. It was observed that autophagy was upregulated in these patients' fibroblasts and proximal tubular cells [40]. The apoptosis rate of proximal renal TECs of cystinosis can be reduced by inhibiting autophagy. Sodium arsenite induced autophagic cell death in renal tubular cells both *in vitro* and *in vivo*, and suppression of autophagy attenuated cell death. Autophagy is also implicated in the cytotoxicity of nanomaterials in proximal tubular cells. Cell death induced by fullerenol exposure at millimolar concentrations was associated with cytoskeleton disruption, autophagic vacuole accumulation, and mitochondrial dysfunction. Furthermore, autophagy inhibitor 3-MA ameliorated loss of mitochondrial membrane potential and ATP depletion, suggesting that autophagy may contribute to fullerenol-induced cell death.

In summary, autophagy is activated in various forms of renal tubular injury. At the current stage, the precise role of autophagy in tubular injury response and the pathogenesis of kidney fibrosis is not well understood. Some studies have investigated how autophagy is involved in the tubule pathogenesis yet findings seemed contradictory. There have been studies that provided evidence to support a cytoprotective role, and others that support deleterious effects of autophagy, which may suggest a context-dependent characteristic. Different types and severity of injuries may produce different outcomes of autophagy. For example, a certain degree of autophagic activity can maintain tissue homeostasis, while higher or excessive autophagic activity leads to apoptosis. Future investigations, for instance, by using targeted autophagic gene knockout mice, are necessary to elucidate the precise functional role of autophagy in tubular injuries.

## 5. Autophagy in Human Immune-Related Renal Diseases

### 5.1. Lupus Nephritis (LN)

**Figure 4:**
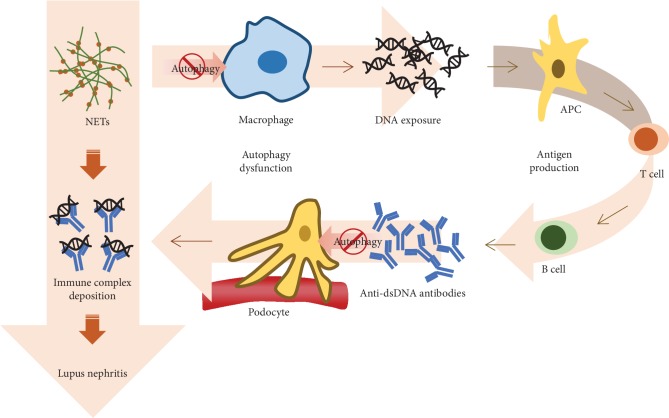
Autophagy dysfunction in lupus nephritis. Macrophagy eliminates neutrophil extracellular trap (NET) through autophagy. Autophagy deficiency in macrophagy leads to DNA exposure of NET, which activates adaptive immunity and produces anti-dsDNA antibodies. Autophagy dysfunction in podocytes fails to clear the autoantibodies, which subsequently binds to DNA fractions of NETs, forming immune complexes. These immune complexes will damage kidney and cause nephritis in lupus.

Systemic lupus erythematosus (SLE; lupus) is a chronic autoimmune disease characterized by production and deposition of autoantibody, damaging multiple tissues and organs. One of the most common and severe complications is lupus nephritis (LN), presenting with proteinuria, hematuria, hypertension, and chronic kidney disease. Early renal involvement of SLE patients often indicates a poor prognosis [41]. More recent studies have revealed that dysregulated autophagy in certain cells are involved in the pathogenesis of LN (Figure 4).

Neutrophils release neutrophil extracellular trap (NET) to restrict the invasion of infectious pathogens. NETs include condensed chromatin and neutrophil proteins, and failing to remove NETs in time might result in autoantibody production. SLE/LN patients exhibited with significant impairment in NET degradation. Consistently, these patients had higher titers of anti-dsDNA antibodies [42]. MTOR inhibitor-treated neutrophils presented with a higher level of autophagy activity and NETs [43]. Thus, we may associate an increased level of autophagy with its impairment in degrading NETs, which may result in the exposure of intracellular antigens to trigger the production of autoantibodies.

A study observed an increased LC3-II expression in spleen and kidney macrophages from lupus mice. Transfer of Beclin-1 knockdown macrophages into macrophage-free lupus mice relieved renal pathological severity, decreased anti-dsDNA titer, and declined urine protein [44]. It indicated that autophagy in macrophage may assist LN development, yet the underlying mechanism deserves further investigation.

As for the renal resident cells, it was reported that podocytes were able to take up SLE patients' anti-dsDNA antibodies, subsequently initiating autophagy to degrade these intracellular aggregations. Chemical inhibition of autophagy in podocytes results in the accumulation of dsDNA antibodies and consequently cell injuries [45]. Likewise, in both lupus mice and human biopsy samples, it was reported that autophagy is only activated in podocytes [46]. Inhibition of autophagy, using 3-MA or ATG5-silencing, resulted in decreased podocyte functioning and increased podocyte layer permeability. Yet, rapamycin treatment to activate autophagy could alleviate podocyte injury.

However, another drug, namely, P140, a spliceosomal peptide, suppresses autophagic flux and increases the LC3 level in B cells. Administration of P140 in lupus-prone mice reduces proteinuria and decreases titer of anti-dsDNA antibodies [47]. Although P140 and rapamycin exhibit opposite effect on autophagy [48–50], they both achieve to attenuate the severity and symptoms of LN. Hence, it indicates that autophagy involves in the development of LN in a complicated way, which is in need of more researches to obtain the full picture and a better target to treat SLE and LN.

### 5.2. IgA Nephropathy (IgAN)

In an earlier paper, it was observed that there were two types of autophagy in the kidney of patients with IgAN [51]. The first type of autophagy, defined as type I autophagy, has a condensed ribosome area with few lipid droplets and a limiting membrane originated from injured mitochondria. Type II autophagy characterized in a condensed ribosome and more lipid droplets, with a limiting membrane from rough ER. In the more recent study, it suggested that type I autophagy occurrence might predict a worse prognosis in IgAN [52]. However, the translational significance of autophagy in renal biopsy still need to be confirmed.

### 5.3. Membranous Nephropathy

In an experimental membranous nephropathy rat model, “passive Heymann nephritis,” ER stress, and autophagy upregulation were observed in podocytes. A recent study also reported that mTORC1 was negatively correlated with autophagy in the passive Heymann nephritis model, where glomerular mTORC1 signaling activation and autophagy downregulation corresponded. Analysis with renal biopsy sample showed increased LC3-positive autophagosomes from patients with membranous glomerulonephritis [19]. ATG3 mRNA was also observed to be significantly higher in microdissected glomeruli from patients with FSGS and membranous glomerulonephritis compared to that in normal controls (pretransplant allograft biopsies).

### 5.4. ANCA-Associated Nephritis (AAN)

ANCA-associated vasculitis (AAV) is an autoimmune disease that targets perivessel tissues and vessel walls of internal organs, characterized with the presence of antineutrophil cytoplasmic antibody (ANCA). The involvement of the kidney in AAV is termed ANCA-associated nephritis (AAN). Patients with AAN often present with hematuria, proteinuria, and cylindruria and might progress rapidly into end-stage renal disease [53]. There were also researches associating AAN with autophagy dysfunction. It was reported that neutrophils treated with ANCA presented with a higher level of autophagy and released more NETs [54]. Anti-LAMP-2 antibody-treated human neutrophils also exhibited with higher activity of autophagy and decreased apoptosis rate. These effects were attenuated by autophagy inhibitors but not by apoptosis inhibitors [55]. Thus, it may be possible that ANCA-stimulated NETosis is closely regulated by autophagy activity.

### 5.5. Diabetic Nephropathy (DN)

Dysregulated autophagy has been suggested to play important pathogenic roles in a variety of disease processes. Inhibition of autophagy was observed in DN rat kidney as well as in type 2 diabetes patient renal biopsy. Possible mechanisms include overactivation of the mTOR pathway, AMPK activation, and decreased expression of silent information regulator 1 (SIRT1). High glucose level mediates the regulation of mTOR, AMPK, and SIRT1, the three nutrient-sensing signal pathways, which all result in the inhibition of autophagy. Inhibition of autophagy might fail to clear AGEs, ROS, or induce ER stress and eventually leads to kidney fibrosis. Therefore, autophagy serves as a protective role in the pathogenesis of DN.

### 5.6. Kidney Transplantation

During and after the transplantation surgery, the donated kidney often faces with various challenges, such as ischemic-reperfusion injury (I/RI) and rejection [56]. Several observations indicate that the kidney exhibited increased activity of autophagy after I/RI. However, it is still under debatable on whether autophagy plays a protective or detrimental role. Neither chemical modulation, such as 3-MA and chloroquine, nor genetic inhibition of autophagy, such as siRNA against *Atg5* and tubule-specific *Atg5* knock-out mice, shows consistent results [32, 35, 56, 57]. One possible reason is that autophagy often displays a dynamic and transient nature, which complicates the comparison between studies where researchers analyzed different durations of I/RI. Moreover, crosstalk of autophagy with other cell death pathways often results in unwanted and even unknown effects. For example, regulating the Bcl-2 protein family not only influences the activity of autophagy but also affects apoptosis since it is also an antiapoptosis protein [58, 59]. Current perspective holds that autophagy switches its role in the development of I/RI, depending on the duration and severity of I/RI [60]. It proposed that in the beginning of I/RI, autophagy is upregulated to exert a protective role against I/RI. When I/RI continues, autophagy increases and reaches a level that starts to contribute to the progress of I/RI. It provides a possible explanation that protective and destructive roles of autophagy are both observed in I/RI, yet further researches are needed to support this hypothesis.

Immune tolerance is another important factor that determines the allograft survival. In general, immunologic tolerance of the allograft includes autoreactive effector T cell deletion and the upregulation of Treg functioning [61]. Pim-2, a Pim family kinase, is found in alloreactive effector T cell and favors its survival and proliferation [62]. It was reported that the Pim-2 level was positively correlated with the severity of allograft rejection and that inhibition of Pim-2 prevented the rejection [63]. Interestingly, T cell regulation, which leads to immune tolerance, is found to be associated with autophagy. In autophagy-deficient mice that follows CD40-CD154 costimulatory blockade, T cell proliferation was enhanced and INF-*γ* production increased, leading to MHC-mismatched allograft rejection [64]. Treatment with rapamycin [65, 66] leads to a higher autophagy level and higher biological quality of Tregs. And higher Treg number is modestly associated with better renal function and lower serum creatinine [66]. It suggests that autophagy regulates T cells to induce immune tolerance and promote allograft survival.

To sum up, in either I/RI or immune rejection that the allograft might face, autophagy achieves to exert its effect, yet its exact role still remains investigation. In current clinical practice, rapamycin, as an autophagy activator, is frequently used to reduce allograft rejection. Hence, autophagy still appears to play a protective role on allograft survival in general. It is possible that autophagy is a double-edge sword in this circumstance and that its protective role outweighs its harmful one, which results in allograft prolonged survival. Nonetheless, researches are needed for further elucidation.

## 6. Perspectives: New Diagnostic and Therapeutic Targets

Undoubtedly, autophagy seems to be a promising target for certain renal disease treatments. There are, however, obstacles that need to be solved before therapy can be used in the clinical setting.

Current autophagy inhibitors, including chloroquine, bafilomycin, rapamycin, and 3-methyladenine, affect more than just autophagy, therefore, it may end up paradoxically to be a deterioration to the sick kidney. More specific and selective autophagy modulator is needed to overcome this dilemma. Moreover, long-term modulation of autophagy might not be as effective and safe as intermittent regulation. The AMPK inducer metformin might meet this requirement, yet inadequate clinical data limits its further application. Another challenging is that the result of modulating autophagy is pretty uncertain, depending on several complex factors such as timing, duration, and intensity of autophagy induction. Under different circumstances, the exact same modulation of autophagy might end in either renal protection or renal cell apoptosis. Therefore, further studies are needed to determine an appropriate condition and therapeutic window where autophagy modulation would yield protective effects. The ability to monitor autophagy in the clinical setting as both a diagnostic tool and a therapeutic guide is still unsolved. Since autophagy is dynamic in its nature, regulated within short-lived and unstable protein-protein binding, it is virtually impossible to capture the dynamic autophagy flux using static biopsy specimens, which is incompatible with routine diagnostic tools.

## 7. Concluding Remarks

In summary, autophagy is activated in various forms of immune-related renal diseases. At the current stage, the precise role of autophagy in renal injury response and the pathogenesis is not well understood. There have been studies that provide evidence to support a cytoprotective role of autophagy, and others that support deleterious effects of autophagy. It is plausible that it is context dependent. The difference in the types of injuries and severity of injuries may produce different outcome of autophagy, in that, a certain degree of autophagic activity can maintain tissue homeostasis, whereas excessive autophagic activity results in cell death. Future investigations, for instance, by using targeted autophagic gene knockout mice, are necessary to elucidate and clarify the precise functional role of autophagy in immune-related renal diseases.

## Figures and Tables

**Table 1:** Autophagy's role in immune cells and renal resident cells.

	Cell type	Role of autophagy	Ref.
Immune cells	Macrophage	Helps control inflammation and contributes to caspase-independent cell death	[6, 7]
	Dendritic cell	Required for virus detection, antigen presentation, and inferon production	[8]
	T cell	Promotes survival and cytokine secretion	[11]
	B cell	Contributes to B cell differentiation and cell death	[12, 13]

Renal resident cells	Podocyte	Autophagy dysfunction is associated with clinical proteinuria and decreased renal function	[17, 24]
	Capillary endothelial cell	Protects endothelial from ROS	[26, 27]
	Mesangial cell	Protects mesangial cell from ROS and contributes to cell survival	[30, 31]
	TEC	Promotes TEC survival and helps eliminate toxins; contributes to renal fibrosis and nephropathic cystinosis	[32, 40]

## References

[1] Mizushima N., Noda T., Yoshimori T. (1998). A protein conjugation system essential for autophagy. *Nature*.

[2] Kim K. H., Lee M. S. (2014). Autophagy—a key player in cellular and body metabolism. *Nature Reviews. Endocrinology*.

[3] Choi A. M., Ryter S. W., Levine B. (2013). Autophagy in human health and disease. *The New England Journal of Medicine*.

[4] Franco L. H., Fleuri A. K. A., Pellison N. C. (2017). Autophagy downstream of endosomal Toll-like receptor signaling in macrophages is a key mechanism for resistance to *Leishmania major* infection. *The Journal of Biological Chemistry*.

[5] Chamilos G., Akoumianaki T., Kyrmizi I., Brakhage A., Beauvais A., Latge J. P. (2016). Melanin targets LC3-associated phagocytosis (LAP): a novel pathogenetic mechanism in fungal disease. *Autophagy*.

[6] Satoh T., Akira S. (2012). Physiological roles and differentiation mechanism of M2 macrophage. *Nihon rinsho. Japanese Journal of Clinical Medicine*.

[7] Qian M., Fang X., Wang X. (2017). Autophagy and inflammation. *Clinical and Translational Medicine*.

[8] Lee H. K., Lund J. M., Ramanathan B., Mizushima N., Iwasaki A. (2007). Autophagy-dependent viral recognition by plasmacytoid dendritic cells. *Science*.

[9] Romao S., Gasser N., Becker A. C. (2013). Autophagy proteins stabilize pathogen-containing phagosomes for prolonged MHC II antigen processing. *The Journal of Cell Biology*.

[10] Puleston D. J., Simon A. K. (2014). Autophagy in the immune system. *Immunology*.

[11] Bronietzki A. W., Schuster M., Schmitz I. (2015). Autophagy in T‐cell development, activation and differentiation. *Immunology and Cell Biology*.

[12] Miller B. C., Zhao Z., Stephenson L. M. (2008). The autophagy gene ATG5 plays an essential role in B lymphocyte development. *Autophagy*.

[13] Hsu H., Xiong J., Goeddel D. V. (1995). The TNF receptor 1-associated protein TRADD signals cell death and NF-*κ*B activation. *Cell*.

[14] Pengo N., Scolari M., Oliva L. (2013). Plasma cells require autophagy for sustainable immunoglobulin production. *Nature Immunology*.

[15] Reiser J., Sever S. (2013). Podocyte biology and pathogenesis of kidney disease. *Annual Review of Medicine*.

[16] Zeng C., Fan Y., Wu J. (2014). Podocyte autophagic activity plays a protective role in renal injury and delays the progression of podocytopathies. *The Journal of Pathology*.

[17] Novelli R., Gagliardini E., Ruggiero B., Benigni A., Remuzzi G. (2016). Any value of podocyte B7-1 as a biomarker in human MCD and FSGS?. *American Journal of Physiology-Renal Physiology*.

[18] Oliva Trejo J. A., Asanuma K., Kim E. H. (2014). Transient increase in proteinuria, poly-ubiquitylated proteins and ER stress markers in podocyte-specific autophagy-deficient mice following unilateral nephrectomy. *Biochemical and Biophysical Research Communications*.

[19] Hartleben B., Gödel M., Meyer-Schwesinger C. (2010). Autophagy influences glomerular disease susceptibility and maintains podocyte homeostasis in aging mice. *The Journal of Clinical Investigation*.

[20] Bechtel W., Helmstädter M., Balica J. (2013). Vps34 deficiency reveals the importance of endocytosis for podocyte homeostasis. *Journal of the American Society of Nephrology*.

[21] Oshima Y., Kinouchi K., Ichihara A. (2011). Prorenin receptor is essential for normal podocyte structure and function. *Journal of the American Society of Nephrology*.

[22] Riediger F., Quack I., Qadri F. (2011). Prorenin receptor is essential for podocyte autophagy and survival. *Journal of the American Society of Nephrology*.

[23] Li C., Siragy H. M. (2017). Autophagy upregulates (pro)renin receptor expression via reduction of P62/SQSTM1 and activation of ERK1/2 signaling pathway in podocytes. *American Journal of Physiology. Regulatory, Integrative and Comparative Physiology*.

[24] Narita M., Young A. R., Arakawa S. (2011). Spatial coupling of mTOR and autophagy augments secretory phenotypes. *Science*.

[25] Yu L., McPhee C. K., Zheng L. (2010). Termination of autophagy and reformation of lysosomes regulated by mTOR. *Nature*.

[26] Chévrier M., Brakch N., Céline L. (2010). Autophagosome maturation is impaired in Fabry disease. *Autophagy*.

[27] Matsuda J., Namba T., Takabatake Y. (2018). Antioxidant role of autophagy in maintaining the integrity of glomerular capillaries. *Autophagy*.

[28] Berman S., Abu Hamad R., Efrati S. (2013). Mesangial cells are responsible for orchestrating the renal podocytes injury in the context of malignant hypertension. *Nephrology*.

[29] Wang S. H., Shih Y. L., Ko W. C., Wei Y. H., Shih C. M. (2008). Cadmium-induced autophagy and apoptosis are mediated by a calcium signaling pathway. *Cellular and Molecular Life Sciences*.

[30] Wang S. H., Shih Y. L., Kuo T. C., Ko W. C., Shih C. M. (2009). Cadmium toxicity toward autophagy through ROS-activated GSK-3beta in mesangial cells. *Toxicological Sciences*.

[31] Ding Y., Kim J. K., Kim S. I. (2010). TGF-{beta}1 protects against mesangial cell apoptosis via induction of autophagy. *The Journal of Biological Chemistry*.

[32] Liu S., Hartleben B., Kretz O. (2012). Autophagy plays a critical role in kidney tubule maintenance, aging and ischemia-reperfusion injury. *Autophagy*.

[33] Periyasamy-Thandavan S., Jiang M., Wei Q., Smith R., Yin X. M., Dong Z. (2008). Autophagy is cytoprotective during cisplatin injury of renal proximal tubular cells. *Kidney International*.

[34] Takahashi A., Kimura T., Takabatake Y. (2012). Autophagy guards against cisplatin-induced acute kidney injury. *The American Journal of Pathology*.

[35] Jiang M., Wei Q., Dong G., Komatsu M., Su Y., Dong Z. (2012). Autophagy in proximal tubules protects against acute kidney injury. *Kidney International*.

[36] Zeng Y., Yang X., Wang J., Fan J., Kong Q., Yu X. (2012). Aristolochic acid I induced autophagy extenuates cell apoptosis via ERK 1/2 pathway in renal tubular epithelial cells. *PLoS One*.

[37] Pallet N., Bouvier N., Legendre C. (2008). Autophagy protects renal tubular cells against cyclosporine toxicity. *Autophagy*.

[38] Kimura T., Takahashi A., Takabatake Y. (2013). Autophagy protects kidney proximal tubule epithelial cells from mitochondrial metabolic stress. *Autophagy*.

[39] Livingston M. J., Ding H. F., Huang S., Hill J. A., Yin X. M., Dong Z. (2016). Persistent activation of autophagy in kidney tubular cells promotes renal interstitial fibrosis during unilateral ureteral obstruction. *Autophagy*.

[40] Sansanwal P., Yen B., Gahl W. A. (2010). Mitochondrial autophagy promotes cellular injury in nephropathic cystinosis. *Journal of the American Society of Nephrology*.

[41] Barnett R. (2016). Systemic lupus erythematosus. *Lancet*.

[42] Hakkim A., Furnrohr B. G., Amann K. (2010). Impairment of neutrophil extracellular trap degradation is associated with lupus nephritis. *Proceedings of the National Academy of Sciences*.

[43] Itakura A., McCarty O. J. T. (2013). Pivotal role for the mTOR pathway in the formation of neutrophil extracellular traps via regulation of autophagy. *American Journal of Physiology Cell Physiology*.

[44] Li B., Yue Y., Dong C., Shi Y., Xiong S. (2014). Blockade of macrophage autophagy ameliorates activated lymphocytes-derived DNA induced murine lupus possibly via inhibition of proinflammatory cytokine production. *Clinical and Experimental Rheumatology*.

[45] Hillmann A., Wardemann H., Pap T., Jacobi A. (2012). Uptake of SLE autoantibodies by podocytes. *Annals of the Rheumatic Diseases*.

[46] Qi Y. Y., Zhou X. J., Cheng F. J. (2018). Increased autophagy is cytoprotective against podocyte injury induced by antibody and interferon-*α* in lupus nephritis. *Annals of the Rheumatic Diseases*.

[47] Page N., Gros F., Schall N. (2011). HSC70 blockade by the therapeutic peptide P140 affects autophagic processes and endogenous MHCII presentation in murine lupus. *Annals of the Rheumatic Diseases*.

[48] Reddy P. S., Legault H. M., Sypek J. P. (2008). Mapping similarities in mTOR pathway perturbations in mouse lupus nephritis models and human lupus nephritis. *Arthritis Research & Therapy*.

[49] Lui S. L., Tsang R., Chan K. W. (2008). Rapamycin attenuates the severity of established nephritis in lupus-prone NZB/W F1 mice. *Nephrology, Dialysis, Transplantation*.

[50] Yap D. Y., Ma M. K., Tang C. S., Chan T. M. (2012). Proliferation signal inhibitors in the treatment of lupus nephritis: preliminary experience. *Nephrology*.

[51] Sato S., Kitamura H., Adachi A., Sasaki Y., Ghazizadeh M. (2006). Two types of autophagy in the podocytes in renal biopsy specimens: ultrastructural study. *Journal of Submicroscopic Cytology and Pathology*.

[52] Sato S., Yanagihara T., Ghazizadeh M. (2009). Correlation of autophagy type in podocytes with histopathological diagnosis of IgA nephropathy. *Pathobiology*.

[53] Jennette J. C., Falk R. J., Hu P., Xiao H. (2013). Pathogenesis of antineutrophil cytoplasmic autoantibody-associated small-vessel vasculitis. *Annual Review of Pathology*.

[54] Sha L. L., Wang H., Wang C., Peng H. Y., Chen M., Zhao M. H. (2016). Autophagy is induced by anti-neutrophil cytoplasmic Abs and promotes neutrophil extracellular traps formation. *Innate Immunity*.

[55] Tang S., Zhang Y., Yin S. W. (2015). Neutrophil extracellular trap formation is associated with autophagy-related signalling in ANCA-associated vasculitis. *Clinical and Experimental Immunology*.

[56] Jiang M., Liu K., Luo J., Dong Z. (2010). Autophagy is a renoprotective mechanism during *in vitro* hypoxia and *in vivo* ischemia-reperfusion injury. *The American Journal of Pathology*.

[57] Kimura T., Takabatake Y., Takahashi A. (2011). Autophagy protects the proximal tubule from degeneration and acute ischemic injury. *Journal of the American Society of Nephrology*.

[58] Chien C. T., Shyue S. K., Lai M. K. (2007). Bcl-xL augmentation potentially reduces ischemia/reperfusion induced proximal and distal tubular apoptosis and autophagy. *Transplantation*.

[59] Isaka Y., Suzuki C., Abe T. (2009). Bcl-2 protects tubular epithelial cells from ischemia/reperfusion injury by dual mechanisms. *Transplantation Proceedings*.

[60] Decuypere J. P., Ceulemans L. J., Agostinis P. (2015). Autophagy and the kidney: implications for ischemia-reperfusion injury and therapy. *American Journal of Kidney Diseases*.

[61] Sun H., Cheng D., Ma Y., Wang H., Liang T., Hou G. (2016). Autophagy in allografts rejection: a new direction?. *Biochemical and Biophysical Research Communications*.

[62] Basu S., Golovina T., Mikheeva T., June C. H., Riley J. L. (2008). Cutting edge: Foxp3-mediated induction of pim 2 allows human T regulatory cells to preferentially expand in rapamycin. *Journal of Immunology*.

[63] Liu H., Zhang C., Liang T., Song J., Hao J., Hou G. (2012). Inhibition of Pim2-prolonged skin allograft survival through the apoptosis regulation pathway. *Cellular & Molecular Immunology*.

[64] Verghese D. A., Yadav A., Bizargity P., Murphy B., Heeger P. S., Schroppel B. (2014). Costimulatory blockade‐induced allograft survival requires Beclin1. *American Journal of Transplantation*.

[65] Akimova T., Kamath B. M., Goebel J. W. (2012). Differing effects of rapamycin or calcineurin inhibitor on T‐regulatory cells in pediatric liver and kidney transplant recipients. *American Journal of Transplantation*.

[66] San Segundo D., Ruiz J. C., Fernández-Fresnedo G. (2006). Calcineurin inhibitors affect circulating regulatory T cells in stable renal transplant recipients. *Transplantation Proceedings*.

